# Accelerating Typhoid Conjugate Vaccine Introduction: What Can Be Learned From Prior New Vaccine Introduction Initiatives?

**DOI:** 10.1093/cid/ciy1118

**Published:** 2019-03-07

**Authors:** Leslie P Jamka, Kenneth W Simiyu, Adwoa D Bentsi-Enchill, Aziza J Mwisongo, Helen Matzger, Anthony A Marfin, Andrew J Pollard, Kathleen M Neuzil

**Affiliations:** 1 Center for Vaccine Development and Global Health at the University of Maryland School of Medicine, Baltimore, MD; 2 Department of Immunization, Vaccines and Biologicals, World Health Organization, Geneva, Switzerland; 3 Center for Vaccine Innovation and Access, PATH, Seattle, Washington; 4 Oxford Vaccine Group, Department of Paediatrics, University of Oxford, and the NIHR Oxford Biomedical Research Centre, Oxford, United Kingdom

**Keywords:** TyVAC, typhoid conjugate vaccine, introduction, Africa, Asia

## Abstract

The health consequences of typhoid, including increasing prevalence of drug-resistant strains, can stress healthcare systems. While vaccination is one of the most successful and cost-effective health interventions, vaccine introduction can take years and require considerable effort. The Typhoid Vaccine Acceleration Consortium (TyVAC) employs an integrated, proactive approach to accelerate the introduction of a new typhoid conjugate vaccine to reduce the burden of typhoid in countries eligible for support from Gavi, the Vaccine Alliance. TyVAC and its partners are executing a plan, informed by prior successful vaccine introductions, and tailored to the nuances of typhoid disease and the typhoid conjugate vaccine. The iterative process detailed herein summarizes the strategy and experience gained from the first 2 years of the project.

Typhoid is a serious enteric fever caused by *Salmonella enterica* subspecies *enterica* Typhi (*S*. Typhi) that is spread through contaminated food, drink, and water. It disproportionately impacts children and marginalized populations in sub-Saharan Africa and Asia [1, 2]. Current estimates indicate that each year there are nearly 12 million cases and >128 000 deaths, with young children and adolescents aged 2–15 years most effected [3]. The high incidence in Asia and Africa [4] coupled with increasing antimicrobial resistance (AMR) of *S.* Typhi have made prevention and control a global health priority [5]. Vaccination and improvements in water, sanitation, and hygiene (WASH) are key to an integrated strategy to control and prevent typhoid.

In late 2016, the Bill & Melinda Gates Foundation funded the Typhoid Vaccine Acceleration Consortium (TyVAC), a partnership between the Center for Vaccine Development and Global Health at the University of Maryland School of Medicine, the Oxford Vaccine Group at the University of Oxford, and PATH, an international nonprofit organization. TyVAC’s goal is to reduce the burden of typhoid and save lives by accelerating the introduction of typhoid conjugate vaccines (TCVs) into Gavi, the Vaccine Alliance–eligible countries. In developing our strategy, we reviewed successful vaccine introductions coupled with the gaps and challenges associated with typhoid and typhoid vaccines.

## SUCCESSFUL VACCINE INTRODUCTION: LEARNING FROM PAST EXAMPLES

**Figure 1.**
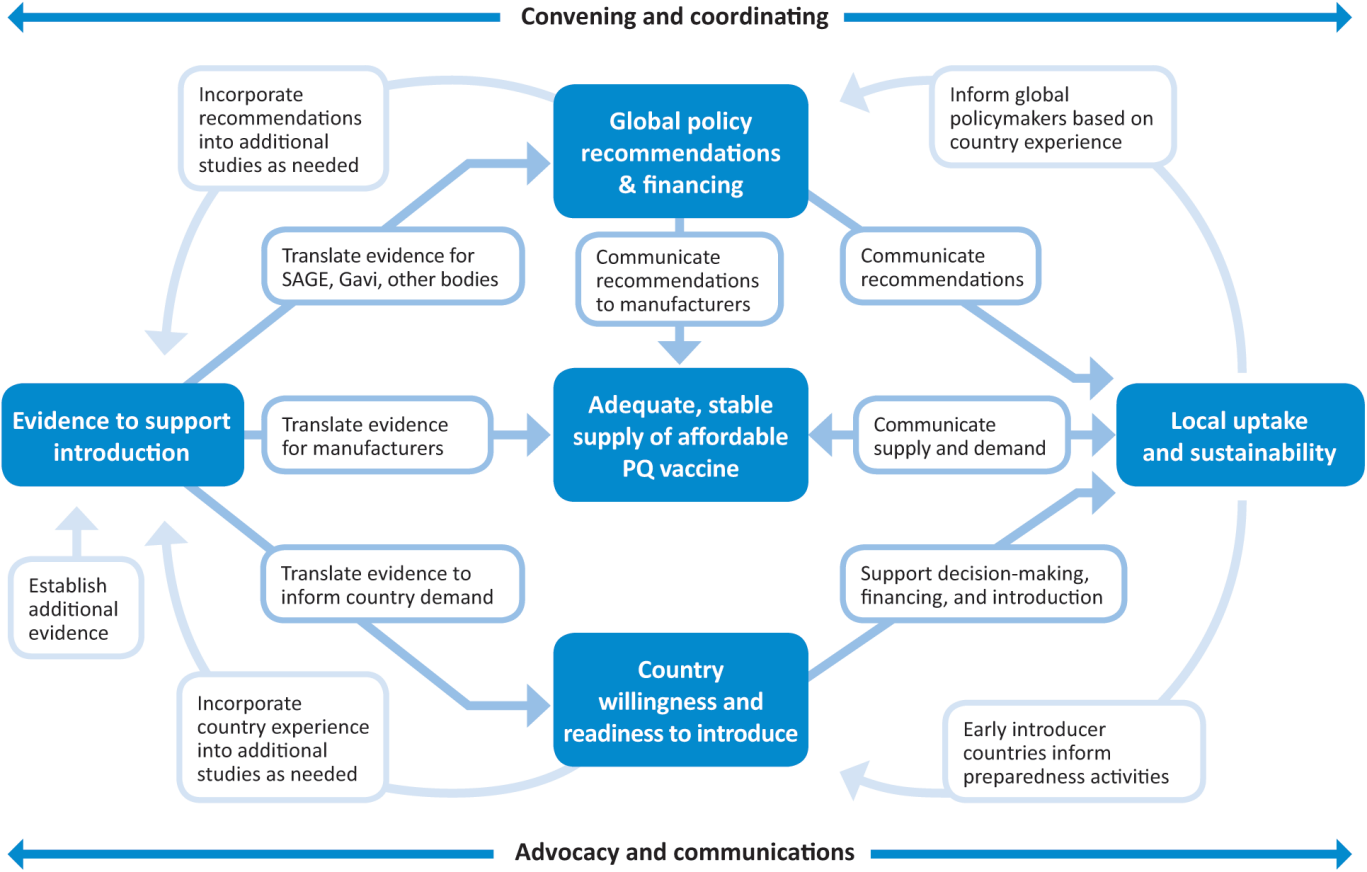
Process to accelerate availability and access to new vaccines. Vaccine introduction is an iterative process, with interactions and feedback loops at each stage. The required components of successful vaccine introductions in low-resource countries are shown in blue boxes, and the interrelationships between stakeholders are shown in the white boxes and with arrows. Previous successful vaccine introduction programs have emphasized the importance of coordination and advocacy and communication across all stages and between stakeholders. Abbreviations: PQ, prequalification; SAGE, World Health Organization Strategic Advisory Group of Experts.

Vaccines are an integral component of global health and disease prevention. “Vaccine introduction” as used in this article refers to the introduction of a *new* vaccine through public health immunization delivery services into national immunization programs. Historically, new vaccine introduction and uptake in developing countries—where they are needed most—has been slow [6–8]. Researchers found that the time from product licensure to Gavi-supported country introduction ranged from .6 to 10.8 years for 6 vaccine-preventable diseases [9]. Since 2000, large-scale initiatives have accelerated a number of vaccine introductions into low-resource populations at the national, regional, and global scales [10]. Based on collective evidence, core elements of Gavi-funded vaccine introduction include (1) evidence on disease burden and vaccine efficacy; (2) licensed and World Health Organization (WHO)–prequalified vaccine; (3) WHO position paper with recommendations for use of vaccine based on research and surveillance data; (4) Gavi board approval to finance vaccine; (5) United Nations Children’s Fund (UNICEF) vaccine tender; and (6) supportive local environments with infrastructure and financing to apply for funding, introduce, and sustain vaccine introduction. While, ideally, vaccine introduction would follow a linear model from evidence to policy to implementation, the reality is that each vaccine introduction is a complex and unique process [11]. Figure 1 illustrates the key components of new vaccine introduction as an iterative process, with feedback loops at every step, and provides a strategic framework for TyVAC. Data are continually generated, processed, shared, and acted upon by international, national, and local stakeholders.

Historical barriers to vaccine introduction include lack of recognition of the value of a vaccine; limited or lack of data, especially on disease burden; weak health systems without systematic decision-making processes, planning, and adequate financing; and lack of global policy recommendations [12, 13]. To inform our approach to TCV introduction, we reviewed the experience of prior vaccine introduction programs. TyVAC’s framework of vaccine introduction (Figure 1) took into account challenges and data gaps from historical vaccine introduction initiatives. Brief summaries of these successful vaccine introduction projects are included to provide the background and basis that paved the way for TyVAC and TCV introduction.

The Meningitis Vaccine Project (MVP) developed, tested, licensed, and introduced MenAfriVac, a group A meningococcal conjugate vaccine, designed for populations in sub-Saharan Africa [14]. A unique challenge facing MVP was the lack of a vaccine suitable for low-income countries, as the available multivalent vaccines were not affordable. MVP ultimately developed a monovalent vaccine, recognizing the lack of a dual market business model (ie, no market in high-resource countries) for this formulation. In addition to the usual challenges of vaccine introduction, MVP had to identify a vaccine manufacturer, coordinate technology transfer, and partner with the manufacturer to develop, test, obtain WHO prequalification, and negotiate an affordable price. The project success was attributed to country and regional engagement, a rigorous clinical development plan, a strategic introduction plan, and key partnerships between countries, international organizations, and the vaccine developer [14–16]. MenAfriVac was prequalified by WHO in June 2010 and introduced the same year. By 2017, >280 million Africans were vaccinated [17].

A comparable example in the Asian region was the Japanese encephalitis (JE) Project, led by PATH and funded by the Bill & Melinda Gates Foundation. JE, a mosquito-borne viral disease that is the leading vaccine-preventable cause of encephalitis in Asia, predominantly affects children in poor, rural communities throughout Asia and the Western Pacific [18]. Severe outbreaks in India and Nepal in 2005 provided visibility for this disease, and an opportunity to introduce JE vaccine into a public health program [19, 20]. Important outcomes from the JE Project included developing guidelines for surveillance and diagnostics; providing relevant data to national and regional entities; establishing good cross-country, regional communication; assessing healthcare infrastructure; enhancing surveillance; and performing close monitoring of vaccine introduction [21]. Subsequently, the JE Project identified an appropriate vaccine for low-income countries; worked with a developing-country vaccine manufacturer to obtain WHO prequalification and supply the vaccine; and formed key partnerships with WHO, Gavi, and UNICEF [22].

Unlike the MenAfriVac and JE examples above, where the burden of disease and need for vaccine is greatest in low- and middle-income countries, for other diseases, considerable burden exists in all countries, and the vaccines are recommended and marketed globally. The Gavi-supported Pneumococcal Vaccines Accelerated Development and Introduction Plan (PneumoADIP), the Rotavirus Vaccine Project (RVP), and the *Haemophilus influenzae* type b (Hib) Initiative are examples of dual-market vaccine programs.

The Hib Initiative leadership recognized that the lack of awareness and data on disease burden, particularly in Asia, were hampering vaccine introduction. Out of the Hib Initiative came a revised WHO policy for vaccine introduction and improved communication on disease burden, vaccine efficacy, safety, and cost-effectiveness [11] and a rapid assessment tool to inform decision making. Gavi support was critical to introduction success [13, 23, 24]. Clear communication about disease burden, financing, and vaccine supply helped accelerate decision making and generate a sense of urgency to support introduction [12]. By the end of 2017, Hib vaccine was introduced in 191 countries [17].

PneumoADIP was designed to address supply and demand issues thought to hinder previous vaccine introductions [6]. The project used a 3-pronged approach of surveillance and research, advocacy and communication (A&C), and vaccine supply and financing [25]. Surveillance studies demonstrated the burden and serotype distribution of disease, providing evidence to underpin vaccine introduction, and groundbreaking A&C efforts brought the importance of pneumonia in under-5 mortality to policymakers, political leaders, and donors. PneumoADIP included an innovative approach using advanced market commitments to secure vaccine manufacturing capacity, a critical obstacle to vaccine supply. By the end of 2017, the estimated global coverage was 44% with pneumococcal vaccine introduced in 136 countries [17].

In 2006, while rotavirus vaccines were licensed and available in high-resource countries, there was no WHO recommendation for use in low-resource settings. Policymakers were uncertain whether rotavirus vaccines would be efficacious in low-resource populations based on clinical studies with prior oral vaccines [26, 27]. In addition to policy, financing, and country introduction work, RVP had to design and execute clinical trials to inform policy and financing decisions. The trials demonstrated that while point estimates of efficacy were lower in developing than industrialized countries, the vaccines prevented more severe disease due to the tremendous burden in those settings; the vaccines were cost-effective and offered “substantial public health benefits” [28–31]. Similar to other vaccine acceleration initiatives, RVP emphasized the importance of stakeholder involvement, unifying and clear communications, logistical feasibility, and political will. The WHO recommended rotavirus vaccines for all countries in 2009. To accelerate vaccine introduction, the Rotavirus Accelerated Vaccine Introduction Network formed in 2016 to provide technical support to countries considering introducing rotavirus vaccines into national immunization programs. As of August 2018, rotavirus vaccines were introduced in 96 countries [32].

## APPLYING LESSONS LEARNED TO TCV

While we have learned a considerable amount from prior successful vaccine introductions, it was equally important to understand typhoid-specific challenges. An influential principle for TyVAC is the recognition, informed by the vaccine introduction projects detailed above, that a WHO recommendation is necessary, but not sufficient, for vaccine introduction at the country level. This principle was a driving factor behind the decision to conduct randomized, placebo-controlled trials, understanding that without information on the impact of vaccine, rather than just immunogenicity and efficacy in a human challenge model, countries will be reluctant to adopt typhoid vaccines [33–36]. TyVAC developed a strategy to overcome challenges and information gaps in 3 main areas: (1) health impact and evidence; (2) global policies, finance, and vaccine supply; and (3) local policies, uptake, and sustainability. Overcoming these challenges is crucial to successful introduction.

### Health Impact and Evidence

 Surveillance and disease burden data are essential to new vaccine introduction. Many countries lack or have limited reliable population-based national burden data that can hamper implementation of preventive measures [37, 38]. For typhoid, assessing the burden of disease at the country level will be particularly challenging due to its unique epidemiologic features. These include its epidemic potential, variability in disease burden and healthcare utilization patterns across settings and over time, and the lack of blood culture availability in many settings.

Disease estimates are often based on a clinical diagnosis of typhoid, which can be confused with other febrile illnesses [2], and the Widal test, which is simple and inexpensive, but frequently inaccurate due to cross-reactivity with other infectious agents. Global agencies are grappling with these challenges, and have emphasized flexibility in defining burden of disease [39, 40]. New WHO surveillance guidelines provide a number of options for countries that are beginning surveillance. Similarly, the Gavi application guidance for typhoid is broad and includes alternative provisions, such as modeling, for countries who do not have robust data on laboratory-confirmed typhoid illness.

### Global Policies, Financing, and Vaccine Supply

 The WHO recommends TCV use: (1) in typhoid-endemic countries as a single routine dose for infants and children aged ≥ 6 months and where feasible, a single catch-up dose for children up to 15 years of age, (2) in response to confirmed outbreaks, and (3) for people at high risk or with high transmission potential [2, 41]. The WHO recommends that typhoid-endemic countries introduce TCV (1) in a single dose for infants and children aged ≥ 6 months and, where feasible, a single catch-up dose for children up to 15 years of age; (2) as a response to confirmed outbreaks; and (3) for people at high risk or with high transmission potential [2, 41]. In November 2017, the Gavi Board approved a US$ 85 million funding window for 2019–2020 to support TCV introduction in developing countries [42].

In formulating the global policy for TCV, the WHO cited immunogenicity data from India [43] as the primary evidence and the high efficacy of a previous TCV in a field trial in Vietnam [44] and TCV efficacy from a controlled human infection model [45] as supporting data. To further quantify vaccine impact, enable cost-effectiveness studies, and foster country-level decision making, TyVAC is conducting vaccine impact studies of Bharat Biotech’s Typbar TCV in Nepal, Bangladesh, and Malawi, and a coadministration immunogenicity study in Burkina Faso [33–36]. The studies are designed to be generalizable to allow data extrapolation to similar epidemiologic settings. The trials in Nepal and Malawi are individually randomized, active-controlled trials that will provide data on culture-confirmed illness and important public health outcomes, including antibiotic usage and hospitalizations. The cluster-randomized study in Bangladesh will provide data on both individual-level impacts on those vaccinated and population-level indirect effects on both those vaccinated and not vaccinated.

In regard to supply, TCV is unlikely to be used in most high-resource settings, except perhaps as a vaccine for travelers. Thus far, there is only one WHO-prequalified TCV. Therefore, vaccine supply and supplier capacity will need to be monitored closely. Assisting with supply–demand estimates is a component of TyVAC [46].

### Local Policies, Uptake, and Sustainability

A TCV program presents numerous challenges to include the poor history with uptake of polysaccharide and oral typhoid vaccines, strained health systems with competing priorities for new vaccines, lack of awareness of the need for a vaccine in addition to WASH and treatment interventions, and inadequate financial sustainability plans at the country level with concerns about affordability. As above, TyVAC is conducting clinical efficacy trials in Bangladesh, Nepal, and Malawi, to provide data in diverse settings on vaccine impact against a number of relevant outcomes. To fill data gaps and inform policy decisions without embarking on new surveillance studies or additional clinical trials, which are not in the funding scope of this project, TyVAC is collecting and collating existing information on typhoid epidemiology and vaccines and conducting systematic reviews of case fatality rates, seasonal patterns, and associations between climatic factors and incidence to model disease burden and impact of vaccine introduction [46]. These efforts complement surveillance projects including the Surveillance of Enteric Fever in Asia Project (SEAP), Severe Typhoid in Africa Program (SETA), and Typhoid Fever Surveillance in Africa Program (TSAP) [38]. SEAP is a large surveillance study collecting data to assess the burden of enteric fever, current trends of AMR of *S*. Typhi, and the cost of typhoid and paratyphoid on families, communities, and healthcare systems in Bangladesh, Nepal, and Pakistan [47, 48]. Similarly, TSAP conducted typhoid surveillance and collected incidence data on typhoid fever in sub-Saharan Africa. Efforts in Africa continue under SETA [49]. Even with these efforts, many countries will have no data. TyVAC is helping to fill this gap through our modeling efforts [50].

AMR of *S*. Typhi is a critical driver of global policy, Gavi financing, and country-specific TCV introduction. Antibiotics are the only effective way to treat typhoid, yet the fast-learning, quick-evolving bacterium has developed defenses against these drugs [51]. Rising fluoroquinolone resistance and emergence of extensively drug-resistant *S.* Typhi strains in Pakistan threaten to spread globally, which makes this a critical time for intervention [52]. Additional information and data on AMR *S*. Typhi and the causes of typhoid, where it is found, and what can be done to stop its spread is available on the Stop Typhoid website. (www.takeontyphoid.org).

## INITIAL STEPS IN COUNTRY-SPECIFIC TCV INTRODUCTION

Successful phase 3 vaccine trials and strong donor support for new vaccine introduction do not guarantee successful vaccine uptake [53]. Other factors are important for country decision making and the initial supplementary immunization activity. Obstacles due to cultural differences, inadequate financing, a poorly planned supply chain, healthcare worker skepticism, lack of parental support, and programmatic suitability can undermine new vaccine introduction if they are not considered and addressed in the planning phase prior to country introduction.

To support successful uptake of TCV, TyVAC identifies concerns, expectations, and roles of ministry of health staff, parents, donors, and healthcare workers. The feasibility and acceptability of a new vaccine, long-term cost, and potential impact of introduction are important functions of today’s ministries of health [54]. Parents have concerns about additional injections, safety, and the need for a new vaccine. Donors want assurances that low-income countries will use a new vaccine and continue to use the vaccine years later when donor financing no longer supports vaccine procurement. Finally, the WHO, as well as global, regional, and national immunization advisors and frontline immunization providers, is aware that adding new vaccines potentially creates new challenges such as concerns about the administration of multiple injections during a vaccination visit [55]. To address these concerns, TyVAC’s broad-based plan integrates country-specific elements of A&C and health economics into engagement with ministries of health, immunization technical advisory groups, national immunization programs, and other stakeholders.

## GETTING THE MESSAGE OUT

Now that global policy recommendations are in place, the TyVAC focus is transitioning to creating messaging, identifying country champions and important national, regional, and local events to connect with the larger typhoid and WASH communities. To generate broader support, we engage, collaborate, and partner with other organizations working on typhoid. In addition, TyVAC identifies opportunities to raise awareness of the global burden by tapping into existing conferences, campaigns, and partner publications to distribute materials, blog, and educate people about typhoid and TCVs.

Leveraging and expanding existing efforts, TyVAC and the Coalition Against Typhoid created a broad call to action to “Take on Typhoid.” The initiative engages a diverse group of stakeholders across the typhoid, vaccines, WASH, and child health sectors to reduce the burden and social impact of typhoid. The website contains advocacy tools, data, and relevant information to ensure partners are informed, empowered, and ready to fight for global, regional, and national prioritization of integrated typhoid control solutions. Resources provide answers to questions, equip champions with the latest data and evidence, and inspire advocacy for stronger WASH and TCV programs. The Take on Typhoid website contains country-specific resources to support country introduction.

## CONCLUSIONS

New vaccine introduction is not a static process and each introduction is unique. Accelerated vaccine introduction requires a strong human component to ensure the necessary relationships and data exchange between global, regional, and local entities, to include policymakers, vaccine developers, researchers, and clinicians. The status of vaccine availability varies and countries have different interests, readiness, willingness, capabilities, and capacities to support introduction. It is important to recognize the need to seek input and achieve buy-in from a broad range of global, regional, and local stakeholders.

TyVAC is fortunate to have the legacy of prior successful vaccine introductions from which to build our strategy. While no approach can guarantee a successful accelerated introduction, the TyVAC process provides a broad schema that includes a diverse array of tools and resources to facilitate introduction. A general framework identifying important components of vaccine introduction vs a step-by-step process has greater transferability to different scenarios. The TyVAC framework of evidence to support TCV introduction, global policy recommendations and financing, country willingness and readiness to introduce, local uptake and sustainability, and adequate, stable supply of affordable vaccine highlights broad components of vaccine introduction used to navigate the intricate process of TCV introduction. Understanding this complexity, level of detail, and uniqueness is critical to accelerating vaccine introduction.

## Notes

***Author contributions.*** All authors read and approved the final manuscript.

***Financial support.*** This publication is based on research funded in part by a grant from the Bill & Melinda Gates Foundation (OPP1151153).

***Supplement sponsorship.*** This supplement is sponsored by the Center for Vaccine Development and Global Health (CVD) at the University of Maryland School of Medicine.

***Potential conflicts of interest.*** A. J. P. has received grants from BMGF, Pfizer, and Okairos; serves as Chair of the UK Department of Health’s Joint Committee on Vaccination and Immunisation and the European Medicines Agency scientific advisory group on vaccines; and serves as a member of the WHO’s Strategic Advisory Group of Experts. K. M. N. is a member of the World Health Organization Strategic Advisory Group of Experts on Immunization. A. D. B.-E. is a staff member of the WHO working on typhoid and typhoid vaccine activities. H. M. has received travel expenses from the University of Maryland; was the Program Officer at BMGF, which made the Typhoid Vaccine Acceleration Consortium (TyVAC) grant; and was Program Officer for the grant for approximately 3 months. All other authors report no potential conflicts. All authors have submitted the ICMJE Form for Disclosure of Potential Conflicts of Interest. Conflicts that the editors consider relevant to the content of the manuscript have been disclosed.
